# Historical and recent processes shaping the geographic range of a rocky intertidal gastropod: phylogeography, ecology, and habitat availability

**DOI:** 10.1002/ece3.1181

**Published:** 2014-07-27

**Authors:** Phillip B Fenberg, Karine Posbic, Michael E Hellberg

**Affiliations:** 1 Ocean and Earth Science, National Oceanography Centre, University of SouthamptonSouthampton, U.K; 2 Department of Biological Sciences, Louisiana State UniversityBaton Rouge, Louisiana

**Keywords:** Baja California, geographic range, habitat availability, historical ecology, *Mexacanthina lugubris lugubris*, museum collections, phylogeography, range limits

## Abstract

Factors shaping the geographic range of a species can be identified when phylogeographic patterns are combined with data on contemporary and historical geographic distribution, range-wide abundance, habitat/food availability, and through comparisons with codistributed taxa. Here, we evaluate range dynamism and phylogeography of the rocky intertidal gastropod *Mexacanthina lugubris lugubris* across its geographic range – the Pacific coast of the Baja peninsula and southern California. We sequenced mitochondrial DNA (CO1) from ten populations and compliment these data with museum records, habitat availability and range-wide field surveys of the distribution and abundance of *M. l. lugubris* and its primary prey (the barnacle *Chthamalus fissus*). The geographic range of *M. l. lugubris* can be characterized by three different events in its history: an old sundering in the mid-peninsular region of Baja (∼ 417,000 years ago) and more recent northern range expansion and southern range contraction. The mid-peninsular break is shared with many terrestrial and marine species, although *M. l. lugubris* represents the first mollusc to show it. This common break is often attributed to a hypothesized ancient seaway bisecting the peninsula, but for *M. l. lugubris* it may result from large habitat gaps in the southern clade. Northern clade populations, particularly near the historical northern limit (prior to the 1970s), have high local abundances and reside in a region with plentiful food and habitat – which makes its northern range conducive to expansion. The observed southern range contraction may result from the opposite scenario, with little food or habitat nearby. Our study highlights the importance of taking an integrative approach to understanding the processes that shape the geographic range of a species via combining range-wide phylogeography data with temporal geographic distributions and spatial patterns of habitat/food availability.

## Introduction

Spatial patterns of gene genealogy can expose patterns hinting at the processes that have shaped the geographic range of species over historic and recent time scales. Reciprocal monophyly between populations may imply vicariance over long historic time scales (say >100 kya, although times will vary with effective population sizes and generation times), whereas more recent processes, such as range expansions, may spread a single allele over a broad range due to founder effects (Hellberg 2014). However, the mere documentation of such patterns tells us little about the underlying physical and biological causes. To better understand the processes that have shaped the geographic range of a species, phylogeographic patterns from a single species should ideally be combined with data on contemporary and historical geographic distribution, range-wide abundance, habitat and food availability, as well as comparisons to codistributed taxa.

Phylogeographic patterns shared by codistributed species may point to major events that have helped shape regional biotas. The mid-peninsular area of Baja California is often identified as a phylogeographic break for many distantly related species, both terrestrial (mammals and reptiles: Riddle et al. 2000; birds, plants and spiders: Zink 2002; Crews and Hedin 2006; Garrick et al. 2009) and marine (fish: Bernardi et al. 2003; Riginos 2005; intertidal isopods: Hurtado et al. 2010; copepods: Peterson et al. 2013). The area encompasses a meeting of temperate and subtropical habitats, both marine and terrestrial, which combine with a complex geological and climatic history (Durham and Allison 1960; Riddle et al. 2000; Case et al. 2002) to create unique habitats with high levels of endemism (Riemann and Ezcurra 2005; Brusca 2010) as well as biogeographic breaks (Riginos 2005; Garrick et al. 2009; Fenberg et al. 2014).

A review of the known locations of phylogeographic breaks in Baja (Munguia-Vega 2011) points to the narrow region between 27.33°N–27.5°N as having the highest concentration for terrestrial species (14 genetic discontinuities). This region coincides with breaks for coastal marine species on the Pacific side of the peninsula near Punta Eugenia and Guerrero Negro Lagoon and at similar latitudes on the Gulf (see above marine references). The most commonly cited reason for this break is the formation of a mid-peninsular seaway during the late Miocene to middle Pleistocene (Upton and Murphy 1997; Riddle et al. 2000; Lindell et al. 2005, 2006; Riginos 2005; Garrick et al. 2009), although geologic evidence for such a seaway remains controversial (Grismer 2002; Hurtado et al. 2010) and estimated dates of divergence vary among studies.

A number marine and terrestrial species found in Baja and southern California also show genetic evidence of a northward range expansion postdating Pleistocene warming (Hellberg et al. 2001; Garrick et al. 2009; Dawson et al. 2010; Haupt et al. 2013; Mantooth et al. 2013). Thus, species with ranges that span the Baja peninsula may exhibit two recognizable genetic signatures from different time scales: a deep mid-peninsular phylogeographic break and a more recent northern range expansion, although the two are rarely mentioned in tandem (but see Garrick et al. 2009 and Mantooth et al. 2013). Far less studied are range dynamics at the southern end of Baja California, where recent climate warming may be expected to have induced range contractions/local extinctions, as observed for species elsewhere (Thomas et al. 2006).

Genetic data from contemporary samples alone cannot reveal a range contraction, which must be elucidated from other sources such as fossils, natural history collections, and field surveys. Yet whether a species is contracting its southern range, expanding its northern range, or both is not entirely dependent upon climate. Probability of range change also depends upon underlying range-wide metapopulation dynamics, size/age structure of populations, local abundances, and habitat and food availability at the leading edges of species ranges (Gilman 2006; Fenberg and Rivadeneira 2011).

Rocky intertidal gastropods with low dispersal potential facilitate tests for the causes underlying phylogeographic breaks and range dynamics for several reasons. First, low dispersal potential increases the probability of preserving historical population subdivision. Second, because rocky intertidal gastropods experience both marine and atmospheric conditions on a daily basis, their ranges may be particularly susceptible to the influence of climate change (Harley et al. 2006). Third, because of their restriction to rocky intertidal habitats and the north–south trending Baja and southern California coastline, the amount and distribution of available habitat within their ranges can be accurately quantified using online mapping tools (e.g. Google Earth). Finally, many Baja and southern California rocky intertidal gastropods are well represented in museum collections and have a detailed history of field surveys, helping to create a baseline of past and present geographic distribution and abundance.

**Figure 1:**
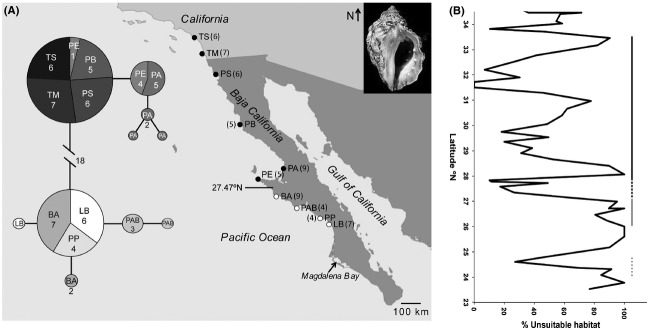
(A) Parsimony network for 10 unique mitochondrial COI haplotypes obtained from 62 *Mexacanthina lugubris lugubris* individuals from the 10 populations. Numbers in parentheses indicate sample sizes from each population. Numbers in the pie charts indicate the number of identical haplotypes from a locality. Sites on the map with white circles belong to the southern clade and sites with black circles belong to the northern clade. The midpoint between sampled northern and southern clades is 27.47°N. TS: Thousand Steps (33.49°N), TM: Tourmaline Beach (32.81°N), PS: Playa Saldamando (31.94°N), PB: Punta Baja (29.96), PA: Playa Altamira (28.53°N), PE: Punta Eugenia (27.82°N), BA: Bahía Ascuncion (27.13°N), PAB: Punta Abreojos (26.70°N); PP: Punta Pequeña (26.23°N); LB La Bocana (26.05°N). (B) Percentage of long stretches of unsuitable habitat (e.g. sandy beach) within successive 48-km (30-mile) bins plotted by latitude across the range of *M. l. lugubris*. A value close to zero indicates that most of the shoreline within that particular bin consists of rocky shore (vice versa for a value near 100). The black line represents the latitudinal span of the northern clade, the grey line is the southern clade, and the white dotted line represents the unsampled region between clades. The grey dotted line represents the latitudinal span of the museum-collected shells outside the modern southern geographic range of *M. l. lugubris* (Fig. 6). Note the general lack of available habitat in the southern clade (11% rocky intertidal) and the ample habitat in the northern range (55% rocky intertidal), particularly at ∼31.5–31.7°N (100% rocky intertidal) where abundances and museum collections are highest (Figs. 5, 6).

The muricid gastropod *Mexacanthina lugubris lugubris* Sowerby, 1821 is found in the high to mid rocky intertidal from southern California to southern Baja California (Fig. 1A), where it primarily preys on the barnacle *Chthamalus fissus* Darwin, 1854 (Marko and Vermeij 1999; Jarrett 2008), although it is also thought to prey upon mussels (*Mytilus californianus*; Becker 2005). *Mexacanthina lugubris lugubris* does not have a planktonic larval stage – rather, juveniles emerge directly from egg capsules attached to rock (Deng and Hazel 2010), hence their dispersal potential is low. Individuals may be locally very common, especially in the northern end of its range.

The range of *M. l. lugubris* does not extend into the Gulf of California, although its sister taxon is the Gulf endemic *Mexacanthina lugubris angelica* (which is now considered to be a separate species from *M. l. lugubris*; Marko and Vermeij 1999; Deng and Hazel 2010). *Mexacanthina lugubris lugubris* is thought to have recently expanded its range from northern Baja to southern California sometime within the past few of decades (see Results and Radwin 1974; Hertz 1995; Becker 2005). Given the high-population genetic structure found in *M. l. angelica* (Deng and Hazel 2010) and other low dispersers along the NE Pacific coast (Hellberg et al. 2001; Pelc et al. 2009; Kelly and Palumbi 2010) and reports supporting a northern range expansion, the potential for observable population genetic subdivision in *M. l. lugubris* and genetic evidence of range expansion is high.

Here, we test for a phylogeographic break in the mid-peninsular region of Baja California and for the genetic signature of a northern range expansion using mitochondrial DNA sequences from *M. l. lugubris* individuals collected from field sites throughout its geographic range. We also look for a potential southern range contraction and assess the underlying physical and biotic causes of range dynamism and phylogeographic patterns by complementing the genetic data with museum records, range-wide field surveys of the distribution and abundance of *M. l. lugubris* and its primary prey (*C. fissus*), habitat availability, and comparative phylogeography.

## Materials and Methods

### Sample collection and sequencing

Adult *M. l. lugubris* individuals were collected from high rocky intertidal habitats between 2003–2007 by PBF at ten localities spanning its full contemporary geographic range (Fig. 1). We preserved specimens in 95% ethanol (at 4°C) and extracted DNA by following the cetyltrimethyl ammonium bromide (CTAB) methods outlined in (Hellberg et al. 2001). We amplified the DNA of 62 individuals using primers HCO1 (Folmer et al. 1994) and LCO1 + (Hellberg et al. 2001) and standard PCR profiles with an annealing temperature of 50°C. Products were directly sequenced in both directions on an ABI sequencer (Applied Biosystems) using the amplification primers.

### Genetic diversity, population subdivision, and population expansion

We constructed a haplotype network using statistical parsimony implemented in the program TCS (Clement et al. 2000). We calculated haplotype diversity for each population using the program DnaSP (Librado and Rozas 2009). We used the program Arlequin 3.5 (Excoffier and Lischer 2010) to calculate nucleotide diversity and mean number of pairwise differences for each population. To test for population subdivision, we performed Analyses of Molecular Variance (AMOVA) using Arlequin 3.5. AMOVAs were first conducted for the northern and southern populations separately. We then performed an AMOVA to compare the variation within and among northern and southern populations. We tested for recent population expansion in the northern populations using mismatch distribution analysis implemented in Arlequin 3.5. Specifically, this analysis generates a distribution of pairwise haplotype differences; a unimodal distribution indicates a population expansion.

### Divergence time estimate

We estimated the time of divergence between the northern and southern populations of *M. l. lugubris* using a coalescent-based method implemented in the program IMa (Hey and Nielsen 2007; Hey 2010). IMa uses Markov Chain Monte Carlo simulations of gene genealogies to estimate the divergence time (*t*), genetic diversities (*θ*_1_, *θ*_2_, and ancestral *θ*), and migration rates (*m*_1_ and *m*_2_) for two populations. To convert the divergence time (*t*), which is scaled by mutation in IMa, to divergence time in years, we used the average COI substitution rate for *Nucella lamellosa* of 7.6 × 10^−9^ substitutions per site per year (McGovern et al. 2010). This in turn scaled to a per locus rate of 4.3 × 10^−6^. We used the infinite-sites model as the substitution model given that the COI sequence alignment showed no sites with more than two variants and the low within-population polymorphism observed. Using the finite-sites model yielded similar results.

We first performed several IMa runs, subsequently adjusting the upper bounds on parameter priors, to determine the most efficient search parameters. We then ran triplicate runs, which differed only in the starting seed. The three runs yielded similar results; we therefore report the results from one run. We recorded the maximum-likelihood estimate from the posterior probability distribution for divergence time and its credibility interval based on the shortest parameter interval containing 90% of the area under the posterior distribution curve. Because the upper end of the posterior probability distribution did not drop to zero, we used the lower bound on the distribution as the parameter value at which the probability dropped to zero at the upper bound (McGovern et al. 2010).

### Field surveys

Presence or absence of *M. l. lugubris* was recorded from at least one field site per latitudinal degree between 23.37°N to 41.74°N from 2002–2007, while PBF sampled for this and a previous study (Fenberg and Rivadeneira 2011). We also collected presence/absence data and assessed the range-wide pattern of abundance of *M. l. lugubris* through a data request from the Pacific Rocky Intertidal Monitoring Program, which includes abundance surveys of NE Pacific invertebrates sampled from 2002–2013 at over 100 field sites from ∼26–58°N, thus spanning nearly the entire contemporary range of *M. l. lugubris*.

Abundance per site was calculated by dividing the number of individuals of each species encountered by the total area surveyed: from 33 quadrats (0.5 × 0.5 m) randomly placed in the high, mid, and low intertidal zone along shore perpendicular transects. A similar sampling method was used for sessile species, which includes the preferred prey species of *M. l. lugubris* – the barnacle *Chthamalus fissus*. The presence of species was recorded underneath 100 points along each transect, from which per cent cover was calculated. Complete sampling protocols may be found at: pacificrockyintertidal.org.

To further confirm the contemporary geographic range of *M. l. lugubris*, we supplemented the above data with temporal presence/absence records of its northern range in southern California obtained from the literature and from its extreme southern range from a rocky intertidal biodiversity survey of Baja (Sagarin et al. 2008; R. Sagarin, pers. comm.).

### Museum collections

We recorded the geographic locality and year of collection for every *M. l. lugubris* shell from the collections within four major museums: the San Diego Museum of Natural History, the Los Angeles County Museum of Natural History, the Santa Barbara Museum of Natural History (online database only), and the Natural History Museum (London). These data were used to set a baseline of the historic geographic distribution of *M. l. lugubris* and to compare similarities between the contemporary range-wide abundance patterns with collection frequency by latitude.

### Habitat availability

We overlaid the geographic range of clades of *M. l. lugubris* onto a plot of habitat availability by latitude using data from Fenberg and Rivadeneira (2011). Briefly, this protocol traces the contours of the coast in Google Earth at a constant elevation of 500 m in cumulative 48 km sections (∼30 miles) while quantifying the proportion within each section that consists of long stretches of sandy beach (>1.6 km; 1 mile) and other unsuitable habitat (e.g. river deltas or lagoons), starting in the south and working our way north. Values close to 100% indicate that a particular 48 km section of coast is composed almost entirely of unsuitable habitat (i.e. not rocky shore).

## Results

### Genetic diversity, population subdivision, and population expansion

**Figure 2:**
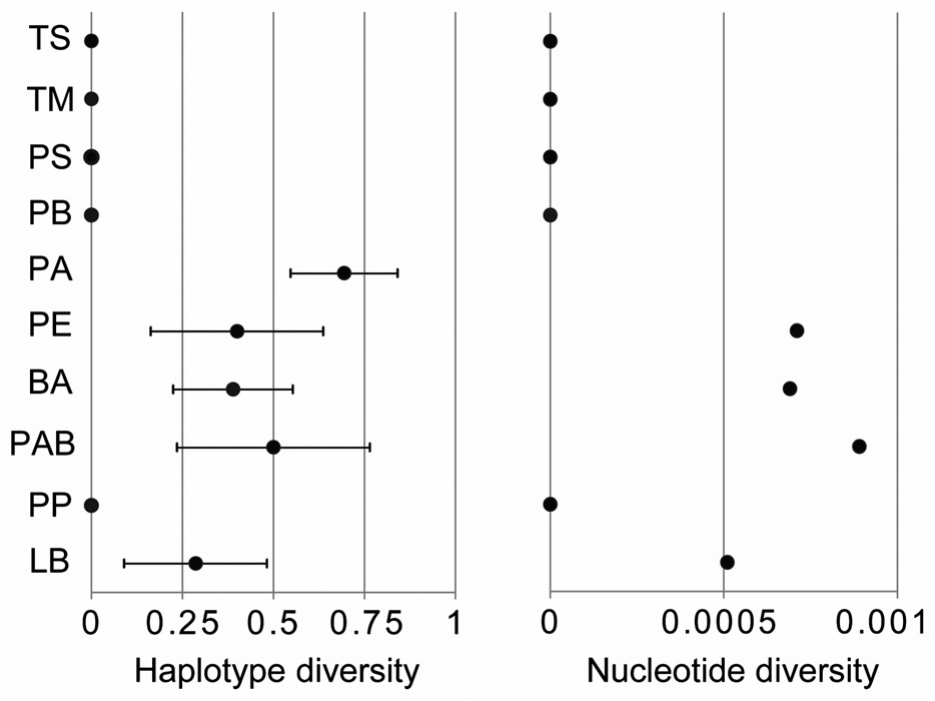
Haplotype and nucleotide diversity for each *Mexacanthina lugubris lugubris* population arranged from top to bottom beginning with the northernmost population. The measure of haplotype diversity ranges from zero (no diversity) to one (most diverse). Bars show standard deviations.

All northern mitochondrial haplotypes are separated from all southern haplotypes by at least 18 mutational steps (Fig. 1). The four northernmost populations have zero diversity while the populations south of Punta Baja show no such latitudinal pattern in genetic diversity (Fig. 2). Overall, however, mean levels of haplotype and nucleotide diversity within the north and south are similar (Table 1).

**Table 1:** Genetic variation and population subdivision in the northern and southern clade. Populations in the northern clade include TS, TM, PS, PB, PA, and PE. Southern clades populations include BA, PAB, PP, and LB (see Fig. 1A)

Population	Haplotype diversity	Mean # pairwise differences	Nucleotide diversity	Variation among populations (%)	Variation within populations (%)
Northern clade	0.52 ± 0.08	0.76 ± 0.57	0.0014 ± 0.001	64	36
Southern clade	0.50 ± 0.12	0.62 ± 0.50	0.0011 ± 0.001	56	44

**Figure 3:**
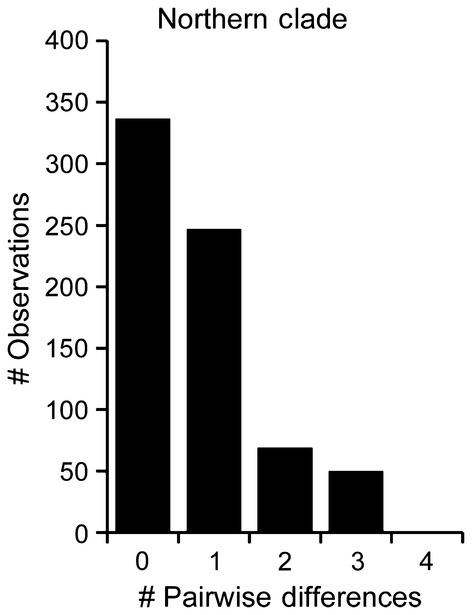
Distributions of pairwise base pair differences in COI sequences between sampled *Mexacanthina lugubris lugubris* individuals in the northern clade.

To test for population subdivision, we performed several AMOVA. When all populations were analysed together, over 95% of the variation observed is explained by differences between the northern and southern regions (Table 2). When the northern and southern populations were analysed separately, the northern populations exhibit a greater variation among populations and less variation within populations compared with southern populations (Table 1). The number of pairwise differences is also similar in northern and southern populations (Table 1). Mismatch analysis of the northern populations yielded a unimodal distribution of pairwise haplotype differences, indicating that the northern populations have undergone a recent expansion (Fig. 3).

**Table 2:** Analysis of molecular variance (Groups = northern and southern clades)

	Per cent variation (%)
Among groups	96
Among populations within groups	2.5

### Divergence time estimate

**Figure 4:**
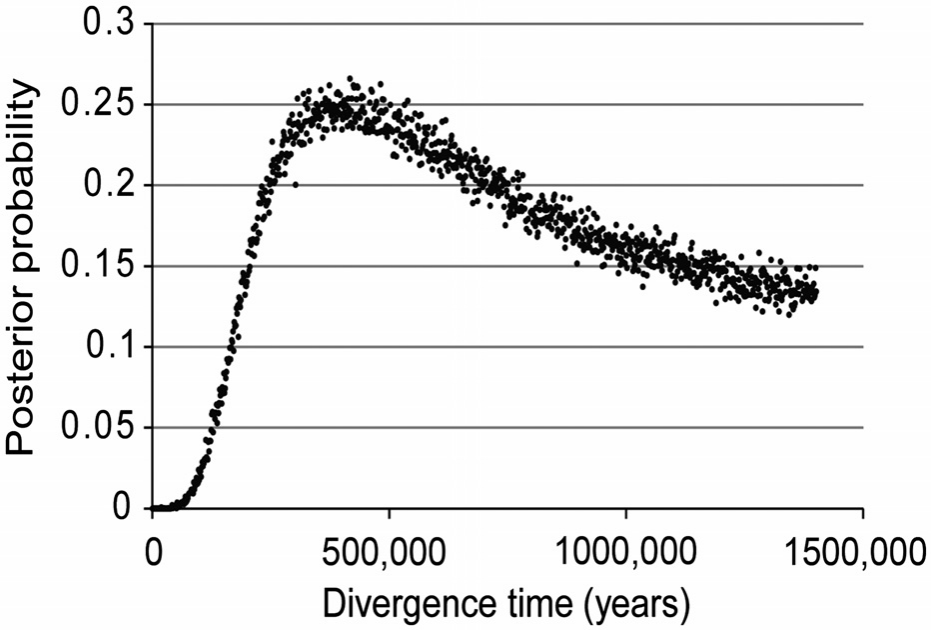
Posterior probability distribution for divergence times (*t*) between northern and southern populations of *Mexacanthina lugubris lugubris*. Divergence time = 417,000 ± 226,000 years ago.

We estimated the time of divergence between northern and southern clade populations using IMa. We found that the northern and southern populations diverged 417,000 ± 226,000 years ago (Fig. 4).

### Field surveys

Every mainland field site (all 21) surveyed between 26.05°N (just north of Magdalena Bay) and 33.54°N (Orange County, CA) revealed the presence of *M. l. lugubris*, thus, we define this latitudinal span as its contemporary geographic range. Reports from professional and amateur collectors and ongoing surveys of Cabrillo National Monument (32.66°N) and surrounding localities indicate that *M. l. lugubris* was absent from the San Diego area for approximately 50 years until it was found at the Monument in 1974 (Radwin 1974; Becker 2005). Twenty to thirty years later, individuals were found further north towards its contemporary range limit (observation by PBF and Hertz 1995), indicating a range expansion of ∼330 km over the last few decades.

At the southern end of the range, a survey of two outer coast sites flanking Magdalena Bay at Cabo San Lazaro (24.8°N) and Punta Tosca (24.3°N) failed to find any individuals during a 2004 expedition (R. Sagarin pers. comm.), nor did PBF and Marko and Vermeij (1999) find any individuals at sites south of Magdalena Bay to the tip of the peninsula at ∼23°N.

**Figure 5:**
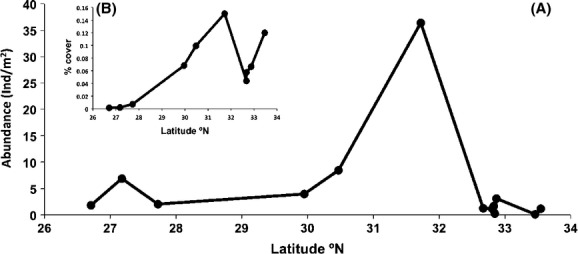
The range-wide abundance pattern (individuals per meter^2^) of *Mexacanthina lugubris lugubris* (A) and the per cent cover of its prey, *Chthamalus fissus*, in the codistributed part of their ranges (B). Both species are most common at 31.7°N (La Bufadora) in northern Baja California.Data from the two sites that were sampled over multiple years are averaged (see text).

The Pacific Rocky Intertidal Monitoring Program surveyed 13 sites spanning nearly the entire range of *M. l. lugubris* (from 26.70°N to 33.54°N) between 2002–2013, with an average abundance of 5.27 (SE = 2.68) individuals per m^2^ (Fig. 5A). Local abundances in the northern range can be very high – 36 individuals per m^2^ were surveyed near Ensenada at La Bufadora (31.72°N). Populations from the southern clade (3 sites) average fewer individuals per m^2^ (3.6, SE = 1.65) than those (10 sites) in the northern clade (5.8 individuals per m^2^, SE = 3.5). For the two northern range sites for which multiple years of data exist (Scripps; 32.87°N and Cabrillo National Monument; 32.67°N), the highest within site densities are from the most recent survey in 2013. For example, *M. l. lugubris* increased in abundance from 0.85 to 2.4 individuals per meter^2^ at Cabrillo National Monument (Zone 1) from 2002 to 2013 (4 years sampled), although the positive relationship is only on the cusp of significance (*R*^2^ = 0.88; *P* = 0.06).

*Mexacanthina lugubris lugubris* is codistributed with its preferred prey, the barnacle *Chthamalus fissus,* throughout its geographic range at least to Punta Abreojos (26.70°N), which is the southernmost sampling locality for the Pacific Rocky Intertidal Monitoring Program. Although the dataset does not distinguish between *Chthamalus* species (*C. fissus* or *C. dalli*), as they are difficult to tell apart in the field, *C. dalli* is not common south of Pt. Conception (Wares and Castañeda 2005), whereas *C. fissus* is common in southern California and Baja (Jarrett 2008). Therefore, we assume that all *Chthamalus* individuals sampled within the range of *M. l. lugubris* are *C. fissus*. The average per cent cover of *C. fissus* for the sites spanning the range of *M. l. lugubris* is 6.2% (SE = 1.6%; Fig. 5B). However, *C. fissus* reaches its highest abundance within this latitudinal span at La Bufadora (31.72°N) in northern Baja, where a per cent cover of 15% was recorded. Abundance patterns in the codistributed span of the ranges of the predatory snail and its barnacle prey are thus similar (Fig. 5).

### Museum collections

**Figure 6:**
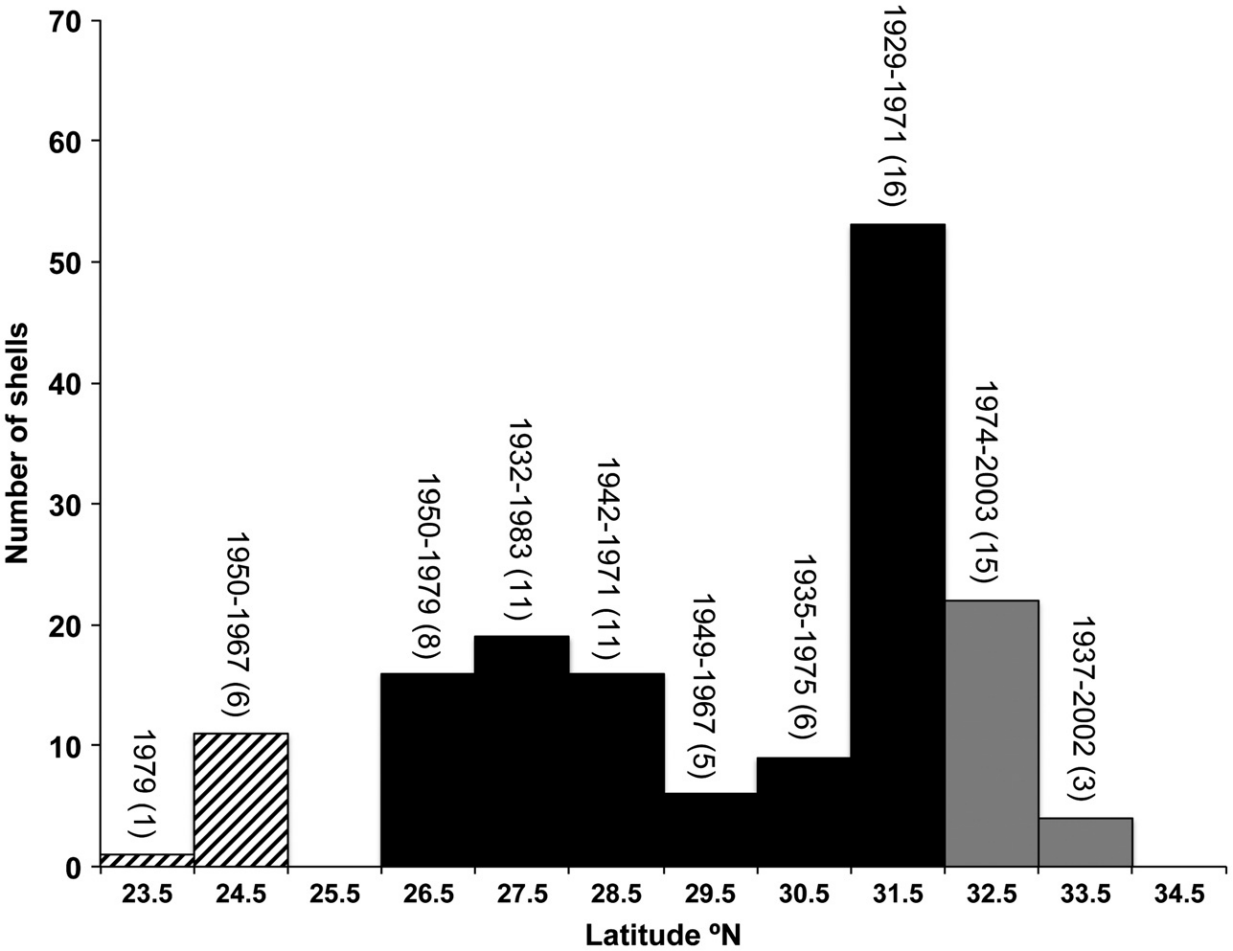
Frequency distribution of museum-collected *Mexacanthina lugubris lugubris* shells from four major natural history museums (see methods). The black bars represent latitudes spanning the modern Baja portion of its geographic range and the grey bars are the expanded range in southern California (NB, three shells from two lots were collected in 1937 and 1955 from 33.5°N, but we cannot discount mislabeling due to the small number). The diagonal striped bars are the latitudes where *M. l. lugubris* was collected outside of its modern southern geographic range. Numbers in parentheses are the number of separate lots per bar (1° bins). The peak in collecting occurred in northern Baja (∼31.5°N), which mirrors its contemporary range-wide abundance pattern (Fig. 5).

The survey of museum collections recorded 82 separate lots containing 157 shells collected between 1929–2003 and spanning 23.95°N to 33.59°N (Fig. 6). The peak in collecting occurs in northern Baja at ∼31.5°N, where 34% (53 shells) of all collected shells (16 lots from 1929–1971) come from, mirroring the latitudinal abundance pattern of *M. l. lugubris,* where the highest abundances are recorded at 31.72°N (Fig. 5A).

The range of years for collections is uneven across the range of *M. l. lugubris*. This is most evident for 32–33°N in southern California, where collections do not include *M. l. lugubris* until 1974 (15 lots of 22 shells; 1974–2002) – a period when most collections of *M. l. lugubris* coincidently stop south of this point (Fig. 6). This pattern cannot be explained by lack of collector effort because codistributed species living in the same habitat were commonly collected here prior to the 1970s. For example, 68 *Lottia gigantea* specimens (11 lots) had been collected in the San Diego vicinity from 1923–1966 for the same museums (data from Fenberg and Rivadeneira 2011). These observations suggest that *M. l. lugubris* was either not common or completely absent from southern California prior to the 1970s. Interestingly, two lots collected in 1937 (1 shell) and 1955 (2 shells) come from Orange County (north of San Diego), suggesting that *M. l. lugubris* may have been locally present in some locations in southern California. However, we cannot judge the robustness of their occurrence here with only two collection lots.

The historical southern range limit as revealed by museum collections is over two degrees further south than what was found recently in the field (26.05°N). Individuals had been collected between 23.9 and 24.8°N from seven lots (12 shells) between 1950–1979 (Fig. 6). We suggest that this spatial discrepancy is real due to the robust locality information for these lots (e.g. “3.5 miles East of Punta Redondo, NW Side of Isla Santa Margarita, Bahia Magdalena”). Interestingly, the individuals collected here may actually be morphologically representative of the previously described *Acanthina tyrianthina* Berry, 1957 (Wu 1985), which is described as lacking a distinct shoulder and with a less frilly aperture compared with *M. l. lugubris*. However, these differences are within the morphological bounds of variation of more northerly collected *M. l. lugubris* shells, thus, *A. tyrianthina* has been defined as a junior subjective synonym (Marko and Vermeij 1999).

### Habitat availability

An average of 50% (SE = 3.3%) of coastline spanning the NE Pacific, from the southern tip of Baja (∼23.5°N) to northern Washington State (∼48.3°N), consists of rocky intertidal habitat. However, the region just south of Punta Eugenia (PE) has the least rocky coastline within this geographic span. Of the ∼1.2° of latitude encompassing the southern clade of *M. l. lugubris*, 89% (SE = 4.5%) consists of sandy beach/unsuitable habitat (i.e. ∼11% rocky intertidal habitat; Fig. 1B). Thus, the southern clade populations inhabit a uniquely sandy section of coast within the range of *M. l. lugubris* and the NE Pacific in general (Fig. 1B). This contrasts with the 55% (SE = 6.4%) of coastline that consists of rocky intertidal habitat in the northern clade, including a large section of coastline (∼100 km) that is composed almost entirely of rocky habitat in northern Baja.

## Discussion

The geographic range of *M. l. lugubris* can be characterized by three different events in its history: an old vicariant event creating a phylogeographic break in the mid-peninsular region of Baja California, a more recent northern range expansion and a southern range contraction. While the cause of the mid-peninsular break remains unknown, the divergence timing we infer supports multiple times of origins for this pattern shared by many taxa. More revealingly, range-wide patterns of distribution and abundance of *M. l. lugubris* and its primary prey (*C. fissus*), along with surveys of habitat and past distributions inferred from museum collections, suggest that the underlying causes of recent range dynamism may be a partial result of the same but opposite trending ecological (local abundances and food availability) and physical (habitat availability) factors at the leading edges of its range.

### Mid-peninsular break

MtDNA sequences from *M. l. lugubris* reveal a phylogeographic break in the mid-peninsular region of Baja California, with populations clearly subdivided into northern and southern clades. The break is located within the 140 km of coastline separating Punta Eugenia and Bahia Ascuncion (Fig. 1A). The mid-point between these sites (27.47°N) lies within the narrow area (27.33°N–27.5°N) that contains the highest concentration of phylogeographic breaks documented for terrestrial species (Munguia-Vega 2011). Mid-peninsular breaks for marine species are often reported in this same vicinity on Pacific and Gulf coasts (Bernardi et al. 2003; Riginos 2005; Hurtado et al. 2010; Peterson et al. 2013).

Despite general agreement on the location of mid-peninsular breaks and its predominance in low dispersing species, disagreement lingers on both the timing and cause of divergence, leading to the possibility of pseudocongruence (Cunningham and Collins 1994). We estimate that northern and southern clades of *M. l. lugubris* diverged approximately 417,000 years ago (±226,000). This range brackets the ∼500 ka divergence estimate (from CO1) described by (Riginos 2005) for several reef fish in the Gulf of California. However, these dates are more recent than those reported for terrestrial species and isopods (around 1 Ma or older: Upton and Murphy 1997; Riddle et al. 2000; Lindell et al. 2005; Garrick et al. 2009; Hurtado et al. 2010).

Likewise, causal mechanisms for this break has been a subject of controversy because the commonly hypothesized vicariant event, a mid-peninsular seaway (Upton and Murphy 1997; Riddle et al. 2000; Riginos 2005), lacks definitive geological or fossil evidence, which has led to debate of its timing, location, extent, and even existence (Grismer 2002; Lindell et al. 2006; Leaché et al. 2007; Hurtado et al. 2010). How such a seaway could induce vicariance for marine species is unclear, as its overall extent and depth have not been surmised. Nonseaway-related hypotheses to explain the shared break for marine species rest on oceanographic differences between the northern to central Baja coast (on both the Pacific and Gulf sides) and the southern part of the peninsula. Along the Pacific side, these regions are characterized by a transition from mid-latitude to tropical conditions, offshore flow of the southward flowing California current, cyclonic eddies north and south of Punta Eugenia, and variation in upwelling regimes (Hewitt 1981; Zaytsev et al. 2003; Herrera-Cervantes et al. 2013). Thus, while a seaway could have caused initial isolation, subsequent genetic homogenization of marine populations would potentially be inhibited by these oceanographic factors. However, because *M. l. lugubris* individuals complete their entire life cycle within the rocky intertidal (i.e. direct development), the probability that oceanographic patterns contributed to their mid-peninsular phylogeographic break is difficult to assess.

We suggest that, for inhabitants of the rocky intertidal, the geographic distribution of available habitat may also impede gene flow between northern and southern regions. We found that the coastline spanning the southern clade of *M. l. lugubris* is the least rocky section of coastline within the NE Pacific. Thus, long distances of unsuitable habitat separate southern Baja populations. For example, southern clade populations at Punta Abreojos and Punta Pequeña are separated by a 100 km stretch of uninhabitable coastline, while individuals at the contemporary southern limit at La Bocana are separated by 150 km of sand from the nearest rocky habitat to the south (Fig. 1). This contrasts with the northern Baja coastline, which includes a continuous stretch of rocky habitat of ∼100 km (Fig. 1B).

We would expect that the long, rock-free distances between southern clade populations would be a historic and ongoing barrier to gene flow for low dispersing Pacific coast rocky intertidal species, such as isopods (Hurtado et al. 2010) and copepods (Peterson et al. 2013). Codistributed species capable of higher dispersal should not exhibit the same pattern, and indeed *Tetraclita rubescens* (3–4 week pelagic larval duration), *Megastraea undosa,* and *Lottia gigantea* (∼5–10 day larval dispersal) exhibit no evidence of a mid-peninsular break (Dawson et al. 2010; Haupt et al. 2013; PBF unpublished).

### Northern range expansion

In contrast to the 18 mutational steps and <140 km separating northern and southern clades, >500 km of coastline in the northern range of *M. l. lugubris* exhibits no CO1 sequence variation (Fig. 2). This pattern, also evident in the unimodal mismatch distribution (Fig. 3), points to a recent northward range expansion. The codistributed but pelagically dispersed snail *M. undosa* shows a similar pattern (Haupt et al. 2013). Both species share dynamic northern range limits, as revealed by their presence in Pleistocene terraces in southern California and subsequent extinction and recolonization (Woodring et al. 1946). Such temporal instability of northern clade populations likely contribute to low genetic diversity in the north, a pattern seen in other rocky intertidal species along the NE Pacific that have undergone northern range change (e.g. Hellberg et al. 2001).

Prior to the 1970s, the northern range limit of *M. l. lugubris* was located near Ensenada (Radwin 1974; Becker 2005), approximately two degrees south of its contemporary northern limit. The shift north is reflected by museum records, which indicate that *M. l. lugubris* was either not common or completely absent from southern California prior to the 1970s. The coastline surrounding Ensenada has the highest contemporary densities of *M. l. lugubris* – 36 individuals per m^2^ were surveyed at La Bufadora (Fig. 5A) in 2003 and (Jarrett 2008) found similarly high local abundances in this vicinity. These high local abundances would provide a large source of individuals fuelling northward expansion. Moreover, over a third of *M. l. lugubris* shells in museum collections come from 31–32°N (Fig. 6), suggesting that *M. l. lugubris* has locally been common in this region for nearly a century.

Ecological factors likely contribute to the high local abundances of *M. l. lugubris* in the northern clade (particularly near Ensenada), including a wealth of habitat and the high cover (15%) of its preferred prey – both *C. fissus* and *M. l. lugubris* reach their highest local abundances across their codistributed ranges here (Fig. 5). Predator-prey dynamics likely contribute to their shared abundance patterns because *M. l. lugubris* is not known to prey extensively on other species (but see Becker 2005). Interestingly, predation pressure from *M. l. lugubris* induces morphological defensive strategies in *C. fissus* by altering the shape of the barnacle's operculum openings (Jarrett 2009). Geographically, narrow or bent operculum *C. fissus* morphs, which are more difficult for *M. l. lugubris* to prey upon, are significantly more common in northern Baja than they are at a site in the expanded range of *M. l. lugubris* (La Jolla – 32.85°N), indicating that high densities of *M. l. lugubris* drives morphology of its prey.

Anthropogenic factors, such as size-selective harvesting of rocky intertidal species for food also influences the abundance of NE Pacific rocky intertidal species (e.g. Lindberg et al. 1998). The human population of northern Baja harvest competitively dominant space occupiers in the mid-high rocky intertidal, such as the owl limpet, *Lottia gigantea* (Pombo and Escofet 1996). Size-selective harvest could allow other, nonharvested species to become more abundant, including *C. fissus* and, indirectly, its predator *M. l. lugubris*. Thus, the anomalously high local abundances and even subsequent range expansion of *M. l. lugubris* may be a partial, albeit indirect, result of human harvesting.

*Mexacanthina lugubris lugubris* joins a growing list of NE Pacific rocky intertidal species expanding their northern ranges in recent decades (Sagarin et al. 1999; Zacherl et al. 2003; Dawson et al. 2010). Consequences of these shifts include the alteration of existing biogeographic classification schemes and the formation of new ecological interactions. However, whether a particular species expands its range depends upon underlying meta-population dynamics, range-wide abundance patterns, and habitat and food availability, which are species specific. *Mexacanthina lugubris lugubris* is conducive to expansion because local abundances at source populations (i.e. northern Baja) are high and it has plenty of habitat and food (*C. fissus*) in this region. In contrast, other NE Pacific rocky intertidal species, such as the pelagically dispersing limpets, *Lottia gigantea,* and *L. scabra*, have low northern range abundances without consistent yearly juvenile recruitment, which in the case of *L. gigantea*, may have contributed to a recent northern range contraction (Gilman 2006; Fenberg and Rivadeneira 2011; Shanks et al. 2014).

### Southern range contraction

As for a number of other species, the southern range limit of *M. l. lugubris* is near Magdalena Bay (Marko and Vermeij 1999; Fenberg and Rivadeneira 2011; Haupt et al. 2013), within a long stretch of coastline with little rocky habitat (Fig. 1B). As a result, the southern range may be less prone to expansion but more prone to contraction. According to museum records (Fig. 6), the southern limit of *M. l. lugubris* once extended past a particularly sandy stretch of coast (∼150 km) to the rocky capes and semiattached islands that flank Magdalena Bay, approximately two degrees south of its current limit. These are the last potential habitat for rocky intertidal species with ranges that predominately extend north. While recent climate warming may underlie the contraction of *M. l. lugubris*, southern populations historically found here would have been tenuous regardless of climate due to their isolation from the nearest population to the north. In addition, food resources appear lacking in this region judging from the low per cent cover of *C. fissus* at sites within the southern clade (Fig. 5B). All of these observations point to the increased probability of Allee effects and eventual range contraction. Thus, not only is *M. l. lugubris* expanding its northern range, but has also contracted its southern range, resulting in an overall northward range shift.

## Conclusions

The geographic range of *M. l. lugubris* can be characterized by three different events in its history: (1) a ∼417,000-year-old split creating northern and southern clades; (2) a more recent northern range expansion; and (3) a southern range contraction. Although solid conclusions about the ultimate cause of the shared mid-peninsular break will remain elusive until more in-depth studies are completed, we suggest that the highly skewed pattern of habitat availability is likely to help maintain divergence in *M. l. lugubris*. Habitat availability appears to be central for explaining recent shifts in range end points in *M. l. lugubris* as well. Ample habitat for *M. l. lugubris* and its food source (*C. fissus*) at the northern range end facilitated high abundance of individuals at source populations, ultimately helping fuel a northward expansion. In contrast, the southern range of *M. l. lugubris* has likely contracted by ∼2° sometime within the past few decades, likely due to a paucity of rocky habitat. Thus, habitat availability appears to be a key factor influencing the past (vicariance) and present (range shifts) geographic range of *M. l. lugubris*.
